# Development and psychometric validation of a brief scale to measure environmental perception based on the 2-major environmental values model in adolescents and adults

**DOI:** 10.1186/s40359-024-01788-5

**Published:** 2024-05-27

**Authors:** Christoph Randler, Talia Härtel, Renan de Almeida Barbosa

**Affiliations:** 1 Eberhard Karls University Tübingen, Auf der Morgenstelle 24, Tübingen, 72076 Germany; 2 Graduate Program in Science Education, Federal University of Rio Grande do Sul, Porto Alegre, Brazil

**Keywords:** Environmental attitudes, 2-MEV model, Brief measurements

## Abstract

**Background:**

The worldwide ecological crisis, including global climate change, is leading to increased awareness and attitudes towards environmental problems. To address these problems, studies of human attitudes are needed. This study is based on the 2-Major Environmental Values (2-MEV) model, which measures two components of environmental attitudes: Preservation and Utilization. The model has been applied to both, adolescents and adults. After decades of use, it is necessary to review the psychometric scale and update the wording. Developing short or even super-short scales to measure well-established constructs is necessary due to time constraints, compliance or fatigue due to language issues.

**Methods:**

We applied an exploratory factor analysis (EFA) to a dataset containing 20 items from the 2-MEV model to reduce the scale to 6 items, 3 per dimension using parallel analysis, scree plot examination and eigen-value greater than 0 as criteria. The scale was then applied to adults and the sample was split for EFA and confirmatory factor analysis (CFA). Multigroup confirmatory factor analysis (MGCFA) was then used to assess invariance across age and gender. Finally, regression and linear models were used to examine the effects of age and gender in both, adults and adolescents.

**Results:**

The 2-MEV model was replicated in the EFA and CFA and the correlated two-factor model showed the best fit. The scale showed configural and metric invariance across age and gender, and scale invariance across gender. Gender and age effects were replicated in relation to previous studies.

**Discussion:**

The brief scale showed good model indices and convergent validity.

**Implications:**

The brief scale of the 2-MEV model can be applied in situations where environmental attitudes are important, but time constraints (internet surveys), compliance, or language problems may hinder the use of longer scales.

**Supplementary Information:**

The online version contains supplementary material available at 10.1186/s40359-024-01788-5.

## Background

The global ecological crisis, including global climate change [1] has led to an increased awareness and attitude toward environmental issues [2]. Addressing these challenges requires a deeper understanding of human perception and attitudes toward the environment. Questionnaires measuring environmental attitudes are essential in identifying patterns of behavior and make it possible to track changes in attitudes over time or across demographic groups. The systematic use of these questionnaires makes it possible to tailor policies and interventions to effectively address environmental challenges [3]. Within the field of social psychology, particularly in the conceptual framework of attitudes, the well-established tripartite model by Eagly and Chaiken [4] categorizes attitudes into three fundamental components: cognitive, affective, and behavioral.

A variety of questionnaires have been developed to reflect environmental attitudes, but a common drawback is there is their specificity to individual studies rather than their global availability. This means that many questionnaires have been designed to meet the specific aims of a single research project, rather than being broadly applicable across different settings, populations or regions [2, 5, 6]. Despite the obvious importance of measuring environmental attitudes, the lack of standardized measurement tools remains a challenge [5, 6]. Among other established scales, such as the New Ecological Paradigm (NEP) [2] the 2-Major Environmental Values (2-MEV) model by Bogner and Wiseman [7] stands out, particularly in its use among adolescents. In the 2-MEV-model, environmental attitude is usually quantified as a self-interested anthropocentric and selfless biocentric domain (e.g., [7–9]). The selfless domain is usually labeled as Preservation (PRE), which is defined as a preference to protect the environment [8]. Antagonistically, the self-interested domain, designated as Utilization (UTL), refers to preferences, such as dominating and exploiting natural resources and the environment. The 2-MEV-model originated from the Environmental Scale developed by Bogner and Wilhelm [10], which measured environmental concern and actual behavior toward the environment using 69 items [11]. Then, the 2-MEV model was further developed to measure environmental attitudes in German speaking populations [12]. The initial validation was based on secondary school students aged 11–18 years. The results fit well into a model of primary factors and two higher-order factors, namely PRE and UTL [7]. The first higher-order factor PRE measures three primary factors: Intent of Support, Care with Resources, and Enjoyment of Nature. The second higher-order factor UTL consists of Human Dominance over Nature and Altering Nature. A high score on the PRE factor indicates an individual who values the conservation and protection of ecological resources, reflecting an ecocentric viewpoint. In contrast, high scores on the UTL factor indicate a more exploitative, anthropocentric perspective towards ecological resources [11, 13]. The 2-MEV model focuses on a PRE and a UTL dimensions, formalized as two independent (orthogonal) facets of the construct ‘environmental attitudes’ [14].

The 2-MEV model has been shown to be robust across different regions and cultures, with validations spanning different continents, over 30 languages and numerous countries, including New Zealand [9], the USA [11], Europe [15, 16], the Ivory Coast [17] and Tanzania [13]. Subsequent validation studies of the 2-MEV have accumulated evidence for its validity from childhood (9–12 years) [11], over adolescents [18, 19], extending into adulthood [20–22]. This supports the assertion that the 2-MEV model is suitable to measure environmental attitudes across age groups and is also culturally invariant and universally applicable.

## Scale evolution is important

After decades of use, it becomes necessary to revisit and update psychometric scales [23]. It’s essential to consider rewording the items as the language used in the questionnaire may become outdated and living conditions may change. For example, since the introduction of the scale in the 1990s world has changed significantly with the advent of the internet and smart phones. Moreover, global environmental awareness has increased, as evidenced by movements such as Fridays for the Future [20] and the growing influence of the Green Party in many countries [24]. These transformative societal shifts underscore the need to reassess and relaunch these scales.

Therefore, Bogner and colleagues consistently revised and adapted the 2-MEV model (e.g., [21, 25]). For example, Kibbe, Bogner [8] proposed negatively coded items to meet the need for psychological testing, although there is still debate as to whether items for the same dimension need to contain both, positively and negatively framed items or whether this question is an extra burden and should just be put to the rest [26, 27]. Further, items should reflect changes in population structure, e.g., in the last decade refugees have arrived in Europe from many countries [28], and adapting scales for simpler and more inclusive language is an important topic.

## Short scales and brief measures are needed

Psychological scales have become longer and longer during earlier decades. With the rise of internet surveys and large-scale studies, psychologist are increasingly opting for shorter scales to ensure the respondent compliance, recognizing that brevity may be more effective [29]. There has been criticism of simply increasing Cronbach’s alpha by including similar questions, as repetitive questions may influence respondents’ answers [30]. The Big Five personality dimensions are designed in various forms to be adaptable to different situations and target populations, thereby increasing their utility (e.g., [31]). Brief measures, such as the Ten-Item Personality Inventory (TIPI) for the Big Five, are valuable in specific contexts, such as online surveys, where compliance is initially high but tends to decline over time [32]. In a study focusing on latecomers to a university lecture, the use of the 10-item TIPI was preferred because of the impracticality of longer questionnaires in this setting [33, 34]. The goal of brief-scale construction is to measure economically with less redundant items while retaining the breadth of the construct [35]. Short measures are effective because they are able to capture essential information efficiently, taking into account time constraints [36, 37]. They also minimize the fatigue and boredom associated with lengthy surveys, thereby improving data quality [38]. Following the precedent of validated short measures in various domains, such as the 10-item Big Five Inventory [33], a short environmental attitude scale can efficiently capture essential dimensions without sacrificing validity or reliability.

## Goals of this study

The aim of this study was to create a condensed brief version of the 2-MEV model for assessing environmental attitudes, with a specific focus on measuring two dimensions through three items each. Three items have been chosen because of a common rule of thumb [39]. Given the established validation of the 2-MEV model spanning from childhood to adulthood, our goal was to develop a version applicable across the entire lifespan. This adaptation would enable the examination of changes in environmental attitudes in both cross-sectional and longitudinal studies. Therefore, measurement invariance across age groups was tested.

Additionally, we aimed to validate the scale’s quality by analyzing age and gender effects. This final step aimed to determine whether the abbreviated measurement could replicate the typical gender and age patterns observed in previous research. As far as we know, our study is the first to test for invariance between groups of participants of different ages, as the 2-MEV is often applied to individuals of the same age group. Here, we present our developmental process in the context of a multi-study research approach.

## Study I: development of a brief measure of the 2-MEV scale

### Methods

First, we reused a data set from a study by Barbosa, Randler [20] where these data have been collected. A total of 327 people from Germany (204 female, 110 male, 3 diverse and 4 preferred not to answer) participated in the survey. Mean age was 23.02 ± 5.14 years (range was 16–61 years). All participants in our study were students at some level of education. In total, 20 participants were from secondary and/or technical education, 145 were undergraduate students and 162 were students pursuing Specialization, Master’s or Doctorate degrees. The participants had to read the invitation with the survey goals, risks, and benefits before answering the questionnaire. Additionally, participants had the option to withdraw from completing the questionnaire at any time (more details, see Barbosa et al. 2021 [20]). The development of the brief measure was based on the 20-item version of the 2-MEV scale provided by Bogner and Wiseman [7]. This scale contains 10 items for UTL and 10 items for PRE (German version: Bogner [40]).

### Statistical analysis

For the statistical analysis of this sample, we used an Exploratory Factor Analysis (EFA) with a principal component extraction and a varimax rotation. To establish the number of factors, we used three criteria: the eigen-value greater than one criterion, the scree plot and a parallel analysis. Reliability analysis was conducted with Cronbach’s α. The parallel analysis was done with an online tool [41]. The parallel analysis was carried out based on 20 variables (items) and 327 cases (participants). All other statistics were carried out with SPSS 28. Regarding missing data, only participants with data for the given variables were retained. For a posthoc power analysis we used the software Webpower [42], and we calculated that with the correlation coefficient, we observed (*r* = .3), at a significance level of 0.01, in a two-sided comparison, with a sample size of 327 observations (participants), the test suggests a power of 0.9987.

## Results

**Fig. 1:**
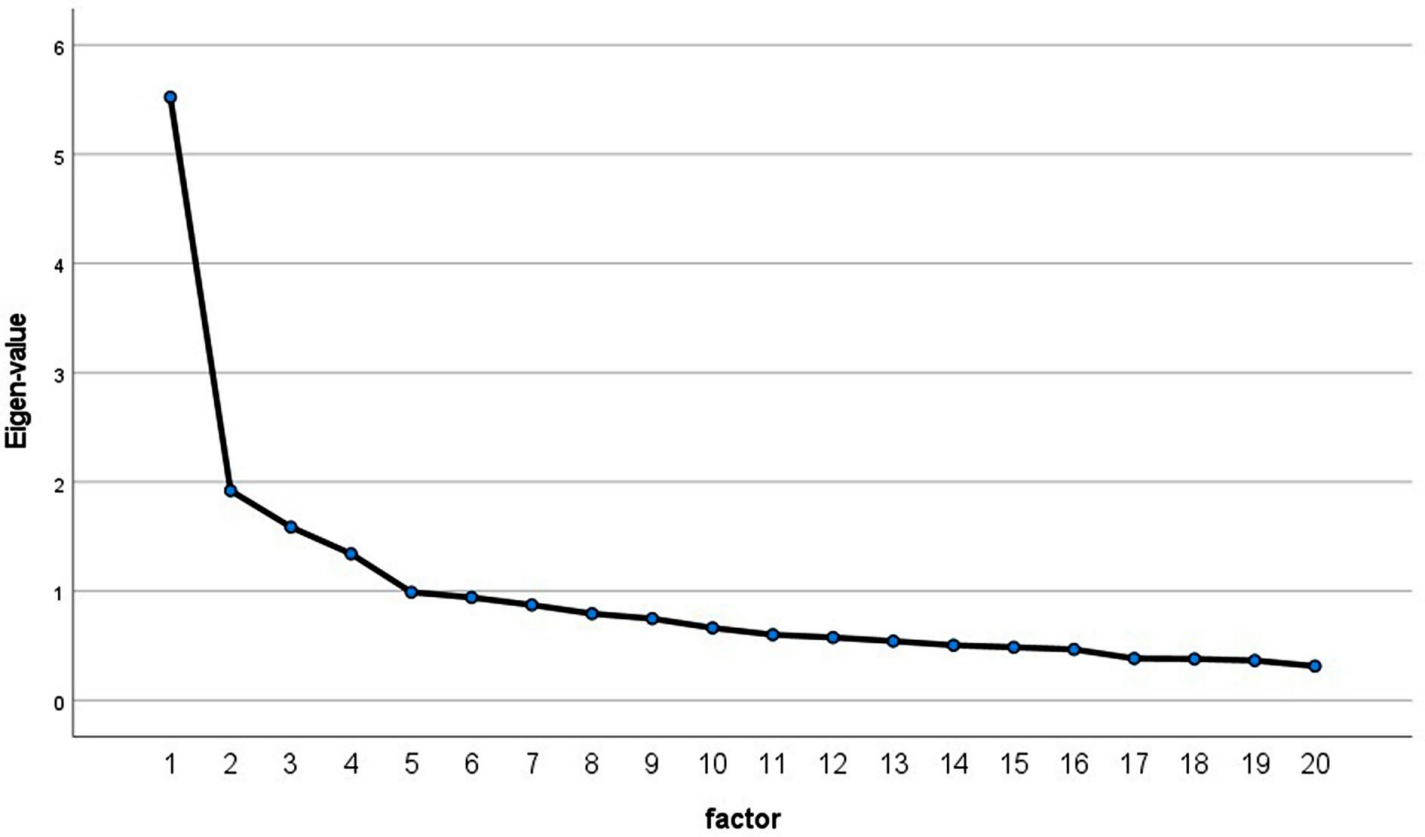
Eigen-values of the exploratory factor analysis

The sample size seems adequate with 1:16, i.e., 16 participants per items [43, 44]. The EFA suggested a four-factor solution (Table 1) based on the eigenvalue greater than one criterion, with the fifth factor below 1.0 (0.990; not shown in Table 1). The parallel analysis supported a three-factor solution with three random eigenvalues lower than the eigenvalues produced by the real data (Table 1). However, the scree plot supports a four-factor solution following the elbow method (Fig. 1).

**Table 1:** Comparison of the factors of the exploratory factor analysis and the parallel analysis based on random eigen-values

Factors	Random eigen-value	Eigen-value	Explained variance (in %)	Cumulative explained variance (in %)
1	1.46	5.52	27.61	27.61
2	1.38	1.92	9.60	37.22
3	1.31	1.59	7.94	45.15
4	1.26	1.34	6.71	51.86

As the results were equivocal, and the 2-MEV scale has been subjected to decades of psychometric testing, which consistently supports a two-factor structure, we set the extraction of factors for the EFA at two factors and rerun the analyses.

The factor loadings after the extraction of two factors and varimax rotation are depicted in Table 2. Kaiser-Meyer-Olkin (KMO)-value was 0.866; Bartlett-test of sphericity was significant (*p* < .001, df = 190, approx. χ2 = 1897.18). The KMO over 0.8 is meritorious (Beavers et al., 2013 [43]). The two factors were labelled in accordance with previous work as Utilization of nature (UTL) and Preservation of nature (PRE) [40].

**Table 2:** Factor loadings according to a two-factor solution of the 2-MEV model (original German items taken from Bogner, 2007). Loadings higher than 0.4 on the respective factor are printed in bold. Communalities are given as h. Items are adopted from ^1^Bogner and Wiseman [7], ^2^Baierl, Kaiser [45], ^3^Baierl, Johnson [46], ^4^Bogner and Wiseman [12], ^5^Bogner and Wiseman [47], ^6^Johnson and Manoli [11], ^7^Baierl, Johnson [48], ^8^Bogner and Wiseman [49], ^9^Bogner and Wiseman [50], ^10^Bogner and Suarez [51], ^11^Schneiderhan-Opel and Bogner [52], ^12^Schneiderhan-Opel and Bogner [53], ^13^Raab and Bogner [54]

	UTL	PRE	h
Human beings are more important than other creatures.^1, 2, 12, 13^	**0.743**	− 0.066	**0.556**
Plants and animals exist primarily to be used by humans.^4, 5^	**0.696**	0.088	**0.492**
Mankind was created to rule over the rest of the nature.^3, 4, 5^	**0.678**	0.105	**0.471**
Humans need not adapt the natural environment, because they can remake it to suit their needs.^2, 5, 11, 12^	**0.589**	− 0.177	0.378
Worrying about the environment often holds up development projects.^1, 2^	**0.552**	− 0.157	0.329
If I get extra pocket money, I will donate a part of it to an environmental organization.^3, 6, 7, 8^	**− 0.547**	0.300	0.390
When I am older, I will actively participate in a nature conservation group.^4, 5, 8, 9^	**− 0.544**	0.465	**0.512**
I often try to persuade others that the environment is an important thing.^4, 5, 8, 9^	**− 0.500**	0.446	**0.448**
Environmental protection costs a lot of money. I am willing to help with a collection. ^3, 4, 5, 9^	**− 0.468**	0.373	0.359
People worry too much about pollution.^2, 10, 11, 12, 13^	**0.457**	− 0.294	0.295
We need to clear forests in order to grow crops.^1, 10, 11, 12, 13^	**0.450**	− 0.244	0.262
We must build more roads so people can travel to the countryside.^1, 7, 11, 12, 13^	**0.409**	− 0.295	0.255
I would really enjoy sitting at the edge of a pond watching dragonflies in flight.^1, 3, 4, 5, 6, 8, 9, 11, 12^	− 0.019	**0.775**	**0.601**
I really like to be able to go on trips into the countryside - for example to forests or fields.^1, 3, 4, 5, 8, 9, 11, 12^	− 0.040	**0.707**	**0.501**
I have a sense of well-being in the silence of nature.^3,4, 5, 8, 9^	− 0.072	**0.690**	**0.482**
It is interesting to know what kinds of creatures live in the lagoons or in the rivers.^10, 12^	− 0.112	**0.586**	0.356
To collect garbage in nature, I would sacrifice my free time.^2, 7^	− 0.355	**0.524**	**0.401**
You are welcome to pick a protected flower if many of them grow in one place.^4^	0.085	− 0.341	0.123
I save water by taking a shower instead of a bath (in order to spare water).^1, 2, 3, 4, 5, 7, 8, 9, 11, 12, 13^	− 0.211	0.286	0.126
Only plants and animals of economical importance need to be protected.^1, 2, 8, 11, 12, 13^	0.217	− 0.245	0.107

Communalities with values of 0.4 or higher are considered for retention, while those below 0.4 are likely to be dropped. According to Costello and Osborne [44] the social sciences, communalities typically fall within the range of low to moderate magnitudes, ranging from 0.4 to 0.7. Concerning factor loadings, Tabachnick and Fidell [55] recommend a minimum threshold of 0.32, while Floyd and Widaman [56] suggest factor loadings exceeding 0.3 and 0.4. Beavers, Lounsbury [43] cited literature that supports factor loadings ranging from 0.6 to 0.7. Items that exhibited factor loadings of 0.3 or higher on two or more factors were classified as “crossloadings” following Costello and Osborne [44]. For a short scale development, we chose the three items per factor with the highest loadings (above 0.6), ensuring cross-loadings remained below 0.3 and communalities are above 0.4. Subsequently, the factor analysis was rerun. KMO-value was 0.710; Bartlett-test of sphericity was significant (*p* < .001. df = 15. approx. χ2 = 519.30). Two factors were extracted exceeded the threshold of the eigen-value greater than one criterion in accordance with a parallel analysis (random eigen-values in brackets): 2.424 [1.185]. 1.676 [1.095]. 0.545 [1.022].

**Table 3:** Factor loadings according to a two-factor solution of the 2-MEV model (original German items taken from Bogner, 2007) based on the reduced set of items from Table 2. Loadings higher than 0.4 on the respective factor are printed in bold. Communalities are given as h

	UTL	PRE	h
Plants and animals exist primarily to be used by humans.	**0.854**	− 0.034	0.730
Mankind was created to rule over the rest of the nature.	**0.844**	− 0.041	0.715
Human beings are more important than other creatures.	**0.768**	− 0.159	0.616
I enjoy trips to the countryside - for example to forests or fields.	− 0.067	**0.836**	**0.704**
I would really enjoy sitting at the edge of a pond watching dragonflies in flight.	− 0.040	**0.834**	**0.697**
I have a sense of well-being in the silence of nature.	− 0.125	**0.790**	**0.639**
*Cronbach’s α*	0.737	0.740	

The EFA shows a clear factor structure with high factor loadings above 0.7 (see Table 3) and very low cross-loadings < 0.16. Although Cronbach’s α was between 0.7 and 0.8, a lower α may be beneficial in a short scale, especially when aiming at measuring a broader construct. Additionally, the small number of items in the scale (*N* = 3) may also contribute to this lower α. Both factors correlated with *r* = .704 with each other. In the next step, a content analysis was conducted, leading to slight modifications in some items to reflect changes in language that have occurred in the last decades. Also, it was checked that all items are useful for different age groups from school students to adults. Some adaptations have been made concerning two items:

The item “I would really enjoy sitting at the edge of a pond watching dragonflies in flight.” (German translation: “Ich sitze gerne am Rande eines Weihers und beobachte dabei zum Beispiel Libellen.“) was changed to a more generic expression “observe nature” rather than using dragonfly/damselfly as an example. We deleted the reference to the insect order Odonata to make the item more suitable on a generic level (without explicitly mentioning a taxon) and to include people who are unable to identify Odonata. This adjustment was made because a recent study showed that the identification of the very common, abundant and widespread Southern Hawker (*Aeshna cyanea*) is not possible in 7th and 8th graders, often not even on the higher taxonomic level of the family Aeshnidae or the order Odonata (see [57]).

The item “Mankind was created to rule over the rest of the nature.” (German translation: “Der Mensch wurde erschaffen, um über den Rest der Natur zu herrschen.”) reads that humans were in some way “created”. This strongly interferes with evolutionary phrasing and may give an illusion of creationism, which should be avoided [58].

### Study II – adult and adolescent sample

#### Methods

The 2-MEV scale was then tested in an adult and an adolescent sample (for 2-MEV scale see Additional file 1). In addition to the scale, we applied a single-item measuring connectedness to nature, which was adopted from Kleespies, Braun [59] (see Additional file 2). To recruit adult participants, an online survey was conducted and distributed across Germany via social media and newsletters of three universities (Tübingen, Bielefeld, Cologne). In addition, participants were invited to take part via a representative online panel. The study was conducted between 25/10/22 and 02/06/23 and the minimum age of participants was 18 years. A total of 3438 people participated in this survey. 1301 were male (38.2%), 2059 female (60.5%), 42 diverse (1.2%) and 36 (1%) did not answer this question. Mean age was 44.14 (SD = 17.01).

To recruit adolescents, a survey was conducted at schools in Germany (federal state of Baden-Württemberg). Students from grade 4 to grade 12 of different school types (primary school as well as medium and higher stratification secondary school) were able to participate. A total of 1752 students participated in the study (813 (46.4%) boys, 910 girls (51.9%), 19 diverse (0.01%). Mean age was 13.1 (SD = 2.58).

We combined the adult survey with the adolescent survey into a combined dataset to analyze gender and age invariance. Measurement invariance analysis was performed to determine whether the structure and parameters of a measurement scale are consistent across different groups [60]. Thus, 5190 people took part, 2114 of whom were male, 2969 female and 61 diverse. Mean Age of the samples are given above.

#### Statistical analysis

The adult sample was randomly split into two, with the first sample used for an EFA, and the second one for a CFA. The EFA sample consisted of 1701 data, and the CFA sample of 1655 data. Further, we compared different measurement models in the CFA: a model with all six items loading onto the same factor, an uncorrelated model with two dimensions (UTL, PRE), and a correlated two-factor model allowing covariance between both factors. We replicated the CFA-model using the adolescent dataset. The following ranges were used for the Fit Indices to indicate a good model fit: Chisquare (χ^2^) – A lower value indicates better fit; Chi-squared *p*-value (χ^2^*p*-value) > 0.05; Minimum Discrepancy Function by Degrees of Freedom divided (CMIN/DFI) < 3; Root Mean Square Residual (RMR) < 0.05; Tucker-Lewis Index (TLI) > 0.95, Comparative Fit Index (CFI) > 0.9, Root Mean Square Error of Approximation (RMSEA) < 0.08; P-value for the test of close fit (PCLOSE) – values close to 1 indicate a good fit [61–63]. Furthermore, the Akaike Information Criterion (AIC) balances the goodness of fit with model complexity. Lower values indicate a better balance [64].

We conducted a Multi-Group Confirmatory Factor Analysis (MGCFA) to determine the best- fitting model of 2-MEV scale was associated with measurement invariance in the German population (*n* **=** 5190). We followed publication standards for a stepwise approach in which the restrictive model (configural invariance) was examined first, followed by increasingly restrictive models with more constrained parameters. This procedure was chosen because it facilitates the identification of parameters of non-invariance in each model [65].

We defined two groups by gender and age to address the MGCFA tests between male (*N* = 2114) and females (*N* = 2969) and adults (*N* = 3438) and adolescents (*N* = 1752). Factorial equivalence was tested between groups. CFA of the 2-MEV model was carried out separately in each group. To assess the MGCFA for testing the measurement invariance, we used the CFI difference test (i.e. ΔCFI ≤ 0.010) following the threshold recommendations of [66]. All statistics were carried out with SPSS 28, the SPSS add-on tool AMOS and R (Version 4.3.2).

## Results

Based on the EFA sample in adults, sampling was adequate (KMO-value = 0.712). Bartlett-test of sphericity was significant (*p* < .001, df = 15, approx. χ2 = 3316.7). Communalities were between 0.66 and 0.78. The eigen-value greater than one criterion suggested a two-factor solution which was supported by parallel analysis (random eigen-values in brackets): 2.53 [1.077]. 1.75 [1.042]. 0.54 [1.013]. The first factor explained 42% of the variance and the second 29%.

Following the criteria mentioned above, the two-factor solution was good with communalities > 0.66; all factor loadings were above 0.8 (see Table 4) and cross-loadings < 0.132 [43, 44]. Both factors exhibited a strong correlation of *r* = .700 with each other.

**Table 4:** Factor loadings according to a two-factor solution of the 2-MEV model (original German items taken from Bogner [40]) based on the reduced set of items from Table 2. Loadings higher than 0.4 on the respective factor are printed in bold. Communalities are given as h

	PRE	UTL	h
I enjoy trips to the countryside - for example to forests or fields.	**0.858**	− 0.096	0.746
I have a sense of well-being in the silence of nature.	**0.835**	− 0.132	0.715
I would really enjoy sitting at the edge of a pond watching nature.	**0.834**	− 0.007	0.695
Mankind should rule over the rest of the nature.	− 0.098	**0.880**	0.785
Plants and animals exist primarily to be used by humans.	− 0.047	**0.822**	0.678
Human beings are more important than other creatures.	− 0.084	**0.809**	0.662
**Cronbach’s α**	0.792	0.787	

We applied a CFA on the adult sample as described in the methods. The fit indices for the three models are shown in Table 5 for adults and in Table 6 for adolescents. Considering these, the correlated two-factor model seems to be a strong contender in both cases. It has a good balance of fit indices, particularly having the lowest AIC, suggesting it may be the most preferable model. Compared to the adults (Table 5), the adolescents show a lower model fit.

**Table 5:** Comparison of different models in the confirmatory factor analysis of the 2-MEV model in adults

	One-factor-model	Uncorrelated two- factor-model	Correlated two-factor-model	Critical values
χ^2^	1272.723	90.957	16.598	Lower = better
df	9	9	8	-
χ^2^*p*-value	< 0.001	< 0.001	0.035	> 0.05
CMIN/df	141.414	10.106	2.075	< 3
TLI	0.316	0.956	0.995	> 0.95
CFI	0.59	0.973	0.997	> 0.95
RMSEA	0.291	0.074	0.025	< 0.08
PCLOSE	0.001	0.002	0.992	Closer 1.0
AIC	1.308.723	114.957	42.598	Lower = better

**Table 6:** Comparison of different models in the confirmatory factor analysis of the 2-MEV model in adolescents

	One-factor-model	Uncorrelated two- factor-model	Correlated two-factor-model	Critical values
χ^2^	810.915	77.327	48.288	Lower = better
df	9	9	8	-
χ^2^*p*-value	< 0.001	< 0.001	< 0.001	> 0.05
CMIN/df	90.102	8.592	6.036	< 3
TLI	0.360	0.946	0.964	> 0.95
CFI	0.616	0.967	0.981	> 0.95
RMSEA	0.229	0.067	0.054	< 0.08
PCLOSE	0.001	0.020	0.288	Closer 1.0
AIC	846.915	113.327	86.288	Lower = better

In adults, connectedness to nature was positively correlated with PRE (*r* = .513. *p* < .001. *N* = 3362) and negatively with UTL (*r* = − .170. *p* < .001. *N* = 3362). The same was found in adolescents (PRE: *r* = .527, *p* < .001, *N* = 1733; UTL: *r* = − .800, *p* < .001, *N* = 1732). This indicates convergent and discriminant validity, respectively. This analysis suggests and supports the two-factor-solution, which is consistent with the theoretical background, the long history of scale’s use and the results of the current analysis.

### Factorial invariance of the short 2-MEV scale across age and gender

**Fig. 2:**
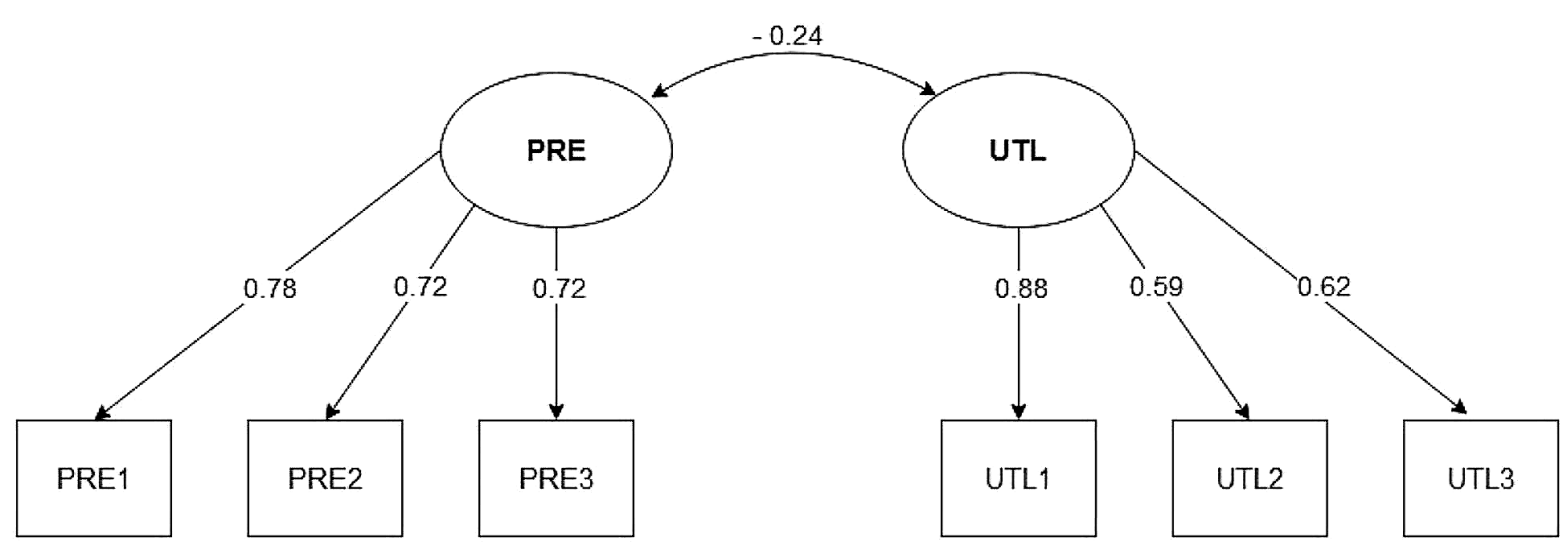
Results of CFA with factor correlations and loadings for the 2-MEV model

The hierarchical factorial model with two sub-factors (UTL and PRE) and one general factor (i.e., Environmental Values) was satisfactory (see Fig. 2).

The configural and metric measurement invariance between males and females was supported according to the set of fit indices assessed (Δχ² = 49.617, Δdf = 16, ΔCFI = 0.996; Δχ² = 75.467, Δdf = 20, ΔCFI = 0.993, respectively, see Table 7). We then proceeded with our stepwise approach and the scalar measurement invariance was supported at the threshold ΔCFI > 0.01 (Δχ² = 134.56, Δdf = 24, ΔCFI = 0.985), but the strict measurement invariance was not tenable.

Configurative and metric measurement invariance between adults and adolescents was supported according to the set of fit indices assessed (Δχ² = 86.739, Δdf = 16, ΔCFI = 0.991; Δχ² = 98.538, Δdf = 20, ΔCFI = 0.990, respectively, see Table 7). Then, we proceeded with our stepwise approach and the scalar and the strict measurement invariance was not tenable (Δχ² = 483.391, Δdf = 24, ΔCFI = 0.944; Δχ² = 1309.416, Δdf = 28, ΔCFI = 0.842).

**Table 7:** Model fit of 2-MEV model CFA and models fit measurement invariance testing with MGCFA across gender and group ages

Model	X^2^	df	CFI	RMSEA	SRMR
Overall Model	50.785	8	0.995	0.032	0.017
**Multigroup Confirmatory Factor Analysis across gender**					
Model	X^2^	df	CFI	RMSEA	SRMR
Male Model	17.19	8	0.997	0.023	0.015
Female Model	32.428	8	0.994	0.032	0.018
Configural Model	49.617	16	0.996	0.029	0.017
Metric Model	75.467	20	0.993	0.033	0.023
Scalar Model	134.56	24	0.985	0.043	0.027
Strict Model	337.947	30	0.96	0.064	0.041
**Multigroup Confirmatory Factor Analysis across age groups**					
Model	X^2^	df	CFI	RMSEA	SRMR
Adults Model	32.087	8	0.996	0.03	0.016
Adolescents Model	54.653	8	0.978	0.058	0.034
Configural Model	86.739	16	0.991	0.041	0.022
Metric Model	98.538	20	0.99	0.039	0.024
Scalar Model	483.391	24	0.944	0.086	0.049
Strict Model	1309.416	30	0.845	0.128	0.077

CFI = comparative fit index; RMSEA = root mean square error of approximation; SRMR = Standardized root mean square residual. Cut-off values for measurement invariance are RMSEA ≤ 0.015, CFI ≤ 0.010, SRMR ≤ 0.25 for loading level and ≤ 0.05 for intercept and residual levels [66, 67]

### Analysis of age and gender effects

**Fig. 3:**
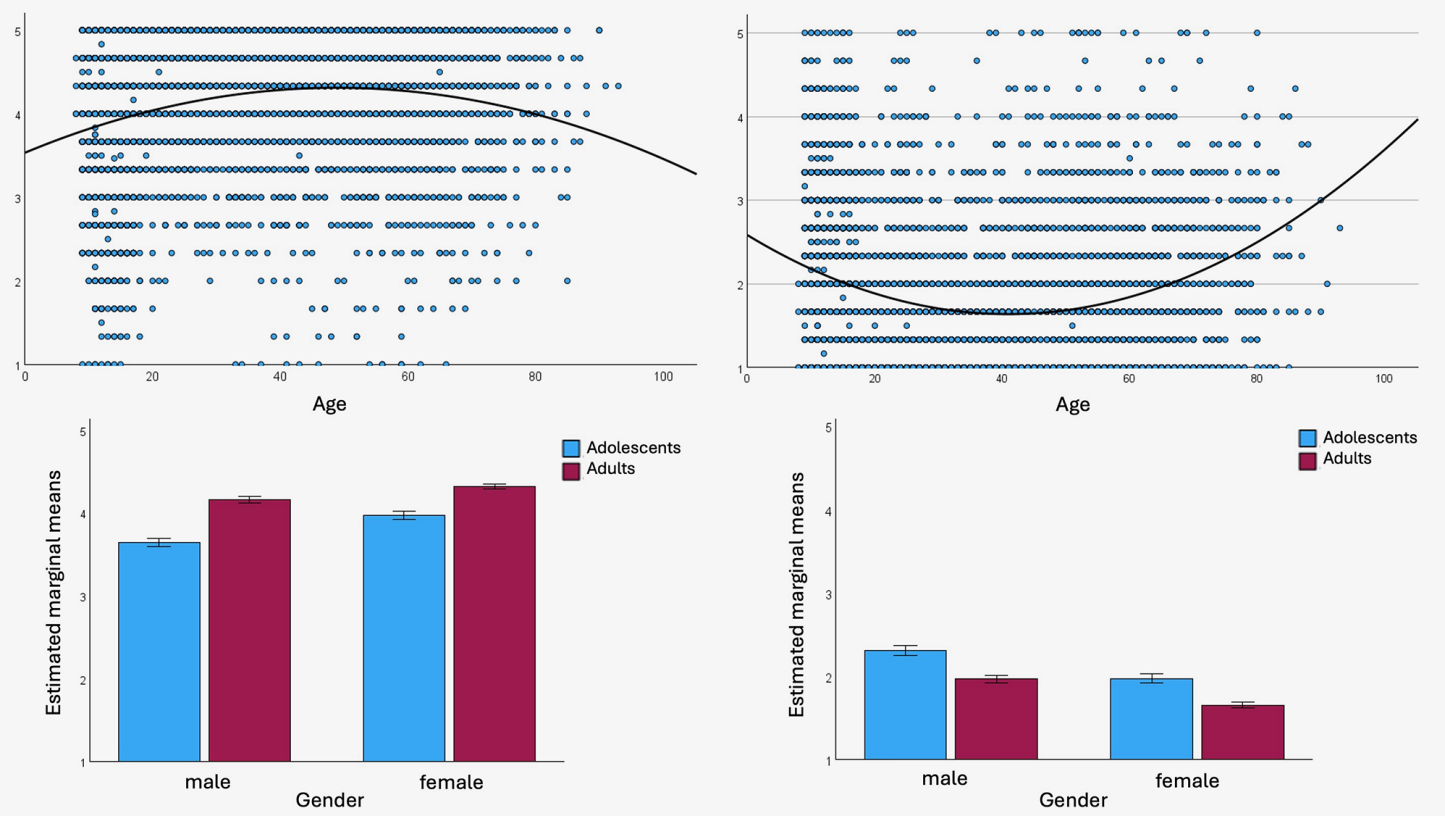
I: Relationship between age in years and (**a**) preservation attitudes (PRE) / (**b**) utilization attitudes (UTL). II: Differences in (**a**) PRE / (**b**) UTL scores across gender (male/female) and age groups (adolescents/adults)

We analysed age and gender effects based on the mean scores per construct, PRE and UTL, in a linear regression with a quadratic term. We found significant effects of age in both measures, PRE and UTL. Both regression models suggested a quadratic relationship between age and PRE or UTL, with an increase in PRE (and decrease in UTL) around the middle-aged years. Thus, the age group around 40–45 years seems the most environmentally concerned age group (Fig. 3 Ia) and Ib)).

In two subsequent linear univariate models based on UTL and PRE as dependent variables we assessed gender and age group as predictors, and including the interaction. Comparing gender and age group revealed significant results for gender and age in both, PRE (Table 8; Fig. 3 – IIa)) and UTL scores (Table 9; Fig. 3 – IIb)). The interaction term gender*age was significant in PRE, but the explained variance (partial eta-squared) was 0.002 and thus, can be considered negligible and irrelevant. Female respondents showed higher PRE and lower UTL scores. Adults showed higher PRE and UTL scores compared to adolescents.

**Table 8:** Analysis of Preservation as dependent variable and age (adult/adolescent) and gender as predictors

Source of variance	df	Mean of squares	F-value	*P*	Partial eta-squared
**Corrected model**	3	95.831	162.954	< 0.001	0.088
**Gender (male/female)**	1	65.092	110.684	< 0.001	0.021
**Age Category (adolescent/adult)**	1	208.826	355.094	< 0.001	0.066
Gender*Age	1	7.396	12.576	< 0.001	0.002
**Error**	5064	0.588			
**Total**	5068				

**Table 9:** Analysis of Utilization as dependent variable and age (adult/adolescent) and gender as predictors

Source of variance	df	Mean of squares	F-value	*P*	Partial eta-squared
**Corrected model**	3	90.748	131.608	< 0.001	0.072
**Gender (male/female)**	1	117.324	170.149	< 0.001	0.033
**Age Category (adolescent/adult)**	1	123.118	178.552	< 0.001	0.034
Gender*Age	1	0.187	0.272	0.602	0.000
**Error**	5059	0.690			
**Total**	5063				

## Discussion

The study presented here shows that the 2-MEV model is a useful tool for measuring the environmental attitudes of adult and adolescent with two factors: Utilization and Preservation. The model where covariance was allowed between the two factors showed the best fit compared to an uncorrelated model or a model where all items are loaded on a single factor. Previous research has shown that the conceptual model developed by Bogner and Wiseman [12] is a good tool for measuring environmental attitudes in adolescents (e.g., [18, 19]), but is also suitable for measuring the construct in adults [20–22].

We have further shown that an updating, rephrasing, and shortening of the scale to a measure with three items per construct provides a valuable tool for addressing these issues. The benefits of this shortened and brief measure are manifold, e.g., including such aspects in research questions where environmental attitudes are not the main issue, and in populations where simple language is advantageous. The brief version is therefore in line with other research that suggests shorter scales, e.g., in personality [68], in well-being and satisfaction of life research [69]. Furthermore, Short scales are valuable in cross-cultural studies where researchers want to ensure that measurements are comparable across different cultural contexts. Brief instruments, such as for self-report measures, can improve the feasibility of cross-cultural research by minimizing respondent burden and increasing participation rates [70]. Especially in longitudinal research, short scales can be advantageous because they help reducing participants fatigue and attrition over time [71].

In particular, the rephrasing of some items to make the wording clearer seems an important aspect [72], for example, removing the special mention of a taxon (Odonata) from the text seems to be an important improvement. Furthermore, in terms of evolutionary biology, the phrase “humankind was created” was rephrased to avoid the term of “creation” to avoid possible interference with evolutionary biology.

We found invariance between genders but not for age groups. The results observed may be due to differential item functioning and do not reflect authentic differences. This suggests that when age groups are compared, these items work differently for adults and adolescents, but not for males and females. This is important for future studies to tackle statistical artifacts and to the scale’s development. Gender differences were similar to other studies with a sufficient sample size, with women showing higher environmental concern [73], and higher attitudes toward animal welfare [74]. This provides additional evidence of the quality of the shortened scale.

While most of the goodness-of-fit measures indicated that configural and metric invariance were met. However, scalar and strict invariance were not fully met, although they were close to the cut-off point between the two genders and between adults and adolescents [66, 67]. This suggests that the difference between groups, particularly between adults and adolescents, was due to a few factor loadings rather than the whole 2-MEV scale [75]. Thus, there was no need to adjust the model. Nevertheless, the differences between age and gender are described as indicative and not conclusive, and future research is needed to examine these differences in new comparative analyses.

The cross-sectional quadratic shape of PRE and UTL cannot be interpreted causally. because it may imply developmental effects, i.e. people in the middle age group may be more environmentally concerned (probably because they are in an age with young children), but it may also be a cohort effect, meaning that people born between 1970 and 1980 are the most environmentally concerned age cohort. Regarding age effects, the characteristic developmental trajectory of adolescents’ environmental attitudes shows an early maximum at around 11 or 12 years of age, followed by a minimum at around 16 years of age [45]. Age does not influence environmental attitudes in adulthood, according to a meta-analysis of [76].

In general, UTL scores are lower compared to PRE scores (e.g., [77, 78]), which is corroborated in our study. Additionally, to the demographic effects of gender and ages which were maintained in our brief instrument, we also provided some convergent validity by using connectedness to nature as another measure. Comparably to other studies [79], we found a positive correlation of connectedness to nature with PRE, and a negative correlation with UTL.

### Limitations

The study was done with a convenience sample of respondents; thus, a representative sample of the German adult population might help to study the scales and the 2-MEV model further.

### Implications

The 2-MEV scale is a well-established measurement instrument with a sound theoretical framework in many cultures since 1994 in Europe to investigate adolescents’ environmental attitudes. Our study supports this view and is intended to increase its usage in the form of a brief scale, that can be applied across the lifespan. Due to its brevity, it can be used even when environmental attitudes are not the main focus of a research study. Another challenge might be the development of a super-short scale with two items, one for PRE and one for UTL. This might be a venue for further research. As there seem differences between adults and adolescents, this points to the importance of environmental education programmes for children and adolescents to create awareness for the environment [80].

### Electronic supplementary material

Below is the link to the electronic supplementary material.

Supplementary Material 1

Supplementary Material 2

## Data Availability

The data that support the findings of this study are available from the authors, but restrictions apply to the availability of these data, which were used under licence for the current study and so are not publicly available. The data are, however, available from the authors upon reasonable request.

## Abbreviations

2-MEV: 2-Major Environmental Values
PRE: Preservation
UTL: Utilization
NEP: New Ecological Paradigm
TIPI: Ten-Item Personality Inventory
EFA: Exploratory Factor Analysis
CFA: Confirmatory Factor Analysis
MGCFA: Multi-Group Confirmatory Factor Analysis
KMO: Kaiser-Meyer-Olkin
CMIN/DFI: Minimum Discrepancy Function by Degrees of Freedom divided
RMR: Root Mean Square Residual
TLI: Tucker-Lewis Index
CFI: Comparative Fit Index
RMSEA: Root Mean Square Error of Approximation
PCLOSE: P-value for the test of close fit
AIC: Akaike Information Criterion
SRMR: Standardized Root Mean Square Residual
