# Supplementary material for: Development and psychometric validation of a brief scale to measure environmental perception based on the 2-major environmental values model in adolescents and adults
Source: BMC Psychol. 2024 May 27;12:300. doi: 10.1186/s40359-024-01788-5 (PMC11131205; doi:10.1186/s40359-024-01788-5)
Supplement: Supplementary file 1 — Supplementary Material 1 [file 40359_2024_1788_MOESM1_ESM.pdf]

2-MEV Scale

English Version

Please indicate the extent to which the following statements apply to you:

	Strongly Disagree	Disagree	Neutral	Agree	Strongly Agree
	1	2	3	4	5
I enjoy trips to the countryside - for example to forests or fields.	[ ]	[ ]	[ ]	[ ]	[ ]
I have a sense of well-being in the silence of nature.	[ ]	[ ]	[ ]	[ ]	[ ]
I would really enjoy sitting at the edge of a pond watching nature.	[ ]	[ ]	[ ]	[ ]	[ ]
Mankind should rule over the rest of the nature.	[ ]	[ ]	[ ]	[ ]	[ ]
Plants and animals exist primarily to be used by humans.	[ ]	[ ]	[ ]	[ ]	[ ]
Human beings are more important than other creatures.	[ ]	[ ]	[ ]	[ ]	[ ]

German Version

Bitte gib an, inwieweit die folgenden Aussagen auf Dich zutreffen:

	trifft überhaupt nicht zu	trifft eher nicht zu	teils/ teils	trifft eher zu	trifft vollkommen zu
	1	2	3	4	5
Tiere und Pflanzen existieren in erster Linie zum Nutzen der Menschen.	[ ]	[ ]	[ ]	[ ]	[ ]
Es macht mir großen Spaß, selbst ins Grüne (Wald, Wiese) hinauszugehen.	[ ]	[ ]	[ ]	[ ]	[ ]
Ich sitze gerne am Rande eines Weihers und betrachte bzw. beobachte dabei die Natur.	[ ]	[ ]	[ ]	[ ]	[ ]
Der Mensch soll über die Natur herrschen.	[ ]	[ ]	[ ]	[ ]	[ ]
Ich fühle mich wohl in der Stille der Natur.	[ ]	[ ]	[ ]	[ ]	[ ]
Menschen sind wichtiger als die anderen Lebewesen.	[ ]	[ ]	[ ]	[ ]	[ ]
