# Supplementary material for: Development and psychometric validation of a brief scale to measure environmental perception based on the 2-major environmental values model in adolescents and adults
Source: BMC Psychol. 2024 May 27;12:300. doi: 10.1186/s40359-024-01788-5 (PMC11131205; doi:10.1186/s40359-024-01788-5)

Connectedness to Nature Item

English Version

Please tick the picture that best describes your connection to nature.

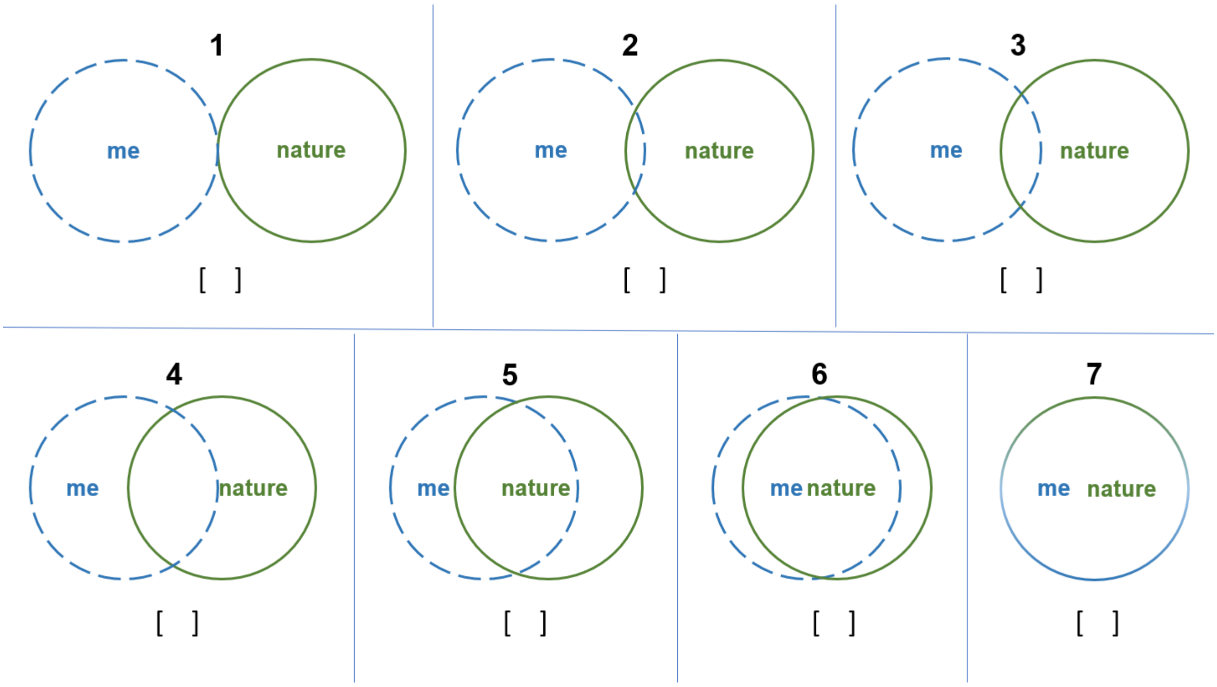

German Version

Bitte kreuze an, welches Bild am ehesten auf Deine Verbindung zur Natur zutrifft.

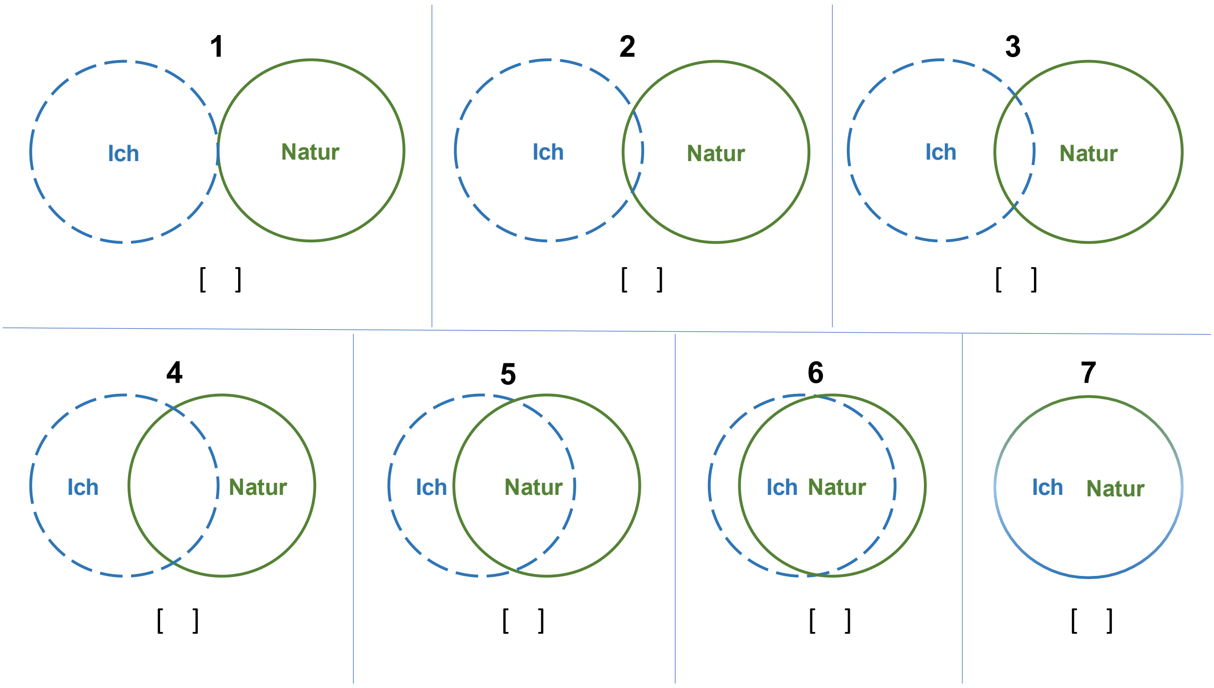

Supplement: Supplementary file 2 — Supplementary Material 2 [file 40359_2024_1788_MOESM2_ESM.pdf]
